# The Indian perspective

**DOI:** 10.1186/1753-6561-7-S5-O1

**Published:** 2013-08-30

**Authors:** MD Gupte

**Affiliations:** 1 ICMR Chair in Epidemiology, Pune, India

## 

The importance and usefulness of cohorts is evident from various studies involving the Framingham cohort in the Unite States. More recently a 50 year follow up study of 18 cohorts has announced a 50% risk of death among those with at least two of the risk factors of hypertension, diabetes, dyslipidemia and smoking. Even though such studies are important the Indian experience with cohorts is limited. This may be due to the relatively less importance of community medicine and public health in the medical education curriculum.

Notable among cohort studies in India were the South Indian BCG vaccination trials and a 15 year study in Chennai to test the efficacy of anti-leprosy vaccines. These studies gave us rich experiences in ensuring community participation. There were pockets of resistance as well as cooperation. There is a need to work and live with the community with sympathy and understanding in order to gain their confidence.

The Indian Council for Medical Research (ICMR) has identified Non-Communicable diseases as a priority area for research and interventions in India. Diabetes and pre-diabetes are rampant in India even affecting those in the 20s. Even though activities and programmes based on population based disease surveillance are going on in several states such as Tamil Nadu and Orissa, we still have a long way to go. Once we get proper surveillance data we can go forward. The ethical implications of establishing and managing biobanks have to be carefully thought out in a country like India with its rich, diverse and sensitive cultural heritage. The Department of Science & Technology has identified some priority areas for research and collaboration with developed countries such as Switzerland and include information & communication technology, materials & nanotechnology, human health sciences, sustainable development and renewable energy. This symposium on cohorts and bio-banks with special reference to non-communicable diseases is part of the broader initiative to promote research and research based initiatives aimed at betterment of the society.

